# The Future State of Doctors

**Published:** 1871-09

**Authors:** 


### The Future State of Doctors.

Terra del Fuego has been traversed by Lieutenant Masters, R. N., who has discovered that the natives believe in devils, and that they are departed spirits of members of the medical profession. The main object of their religious ceremonies is to keep these devils at a distance from them.—*Times and Gaz.*

Let the Lieutenant tell that to the marines.—*Clinic.*

